# Supplementary figures and images for: Cell type-specific weighting-factors to solve solid organs-specific limitations of single cell RNA-sequencing
Source: PLoS Genet. 2024 Nov 18;20(11):e1011436. doi: 10.1371/journal.pgen.1011436 (PMC11573148; doi:10.1371/journal.pgen.1011436)

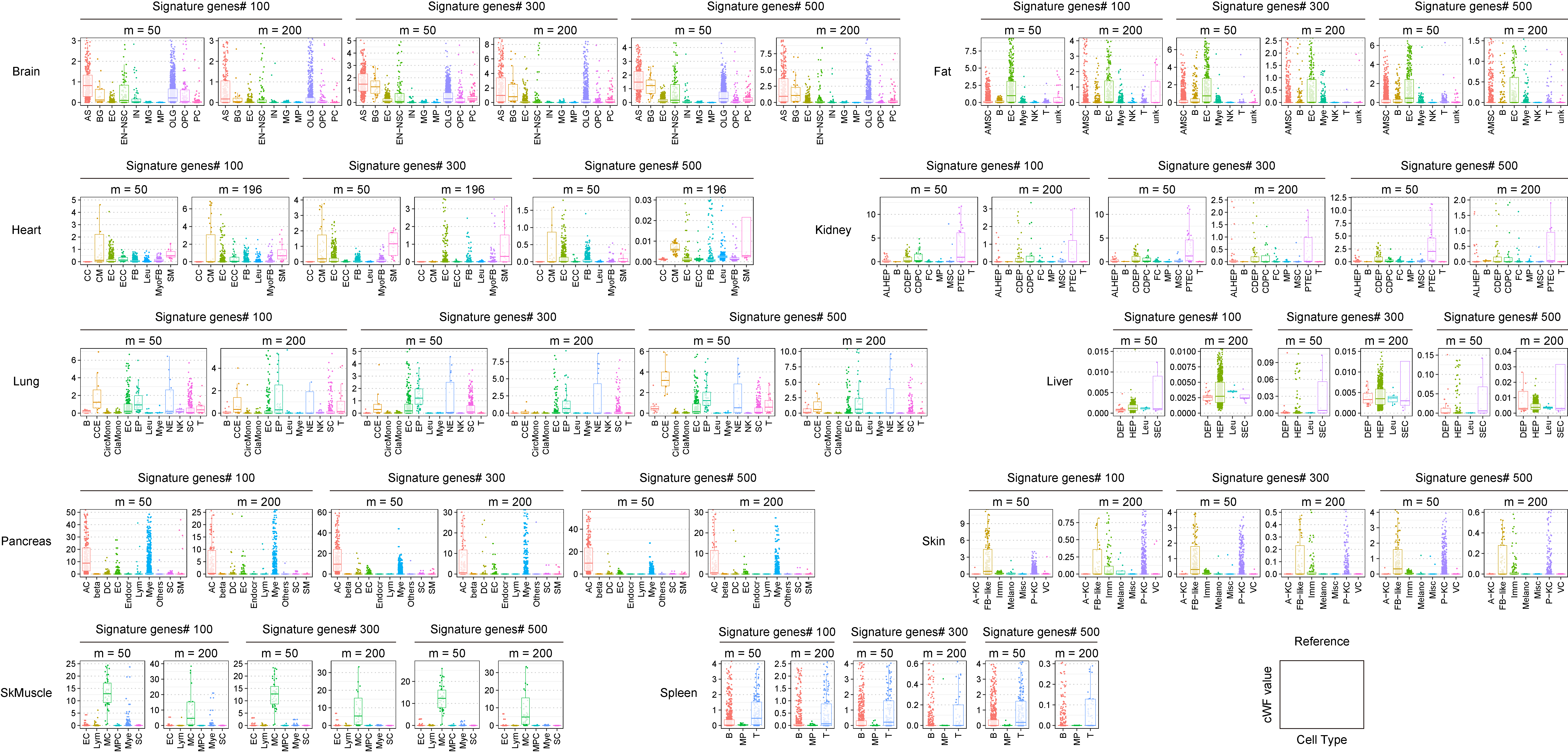

Supplement: S1 Fig — The cWFs for each cell type of each organ are shown for the m = 50 and m = 200. The results are shown for the signature genes numbers (Signature genes#), 100, 300, and 500. Compare the results to those with the default m = 100 (Fig 3). Raw data are available as S3 Table. (TIF) [file pgen.1011436.s001.tif]

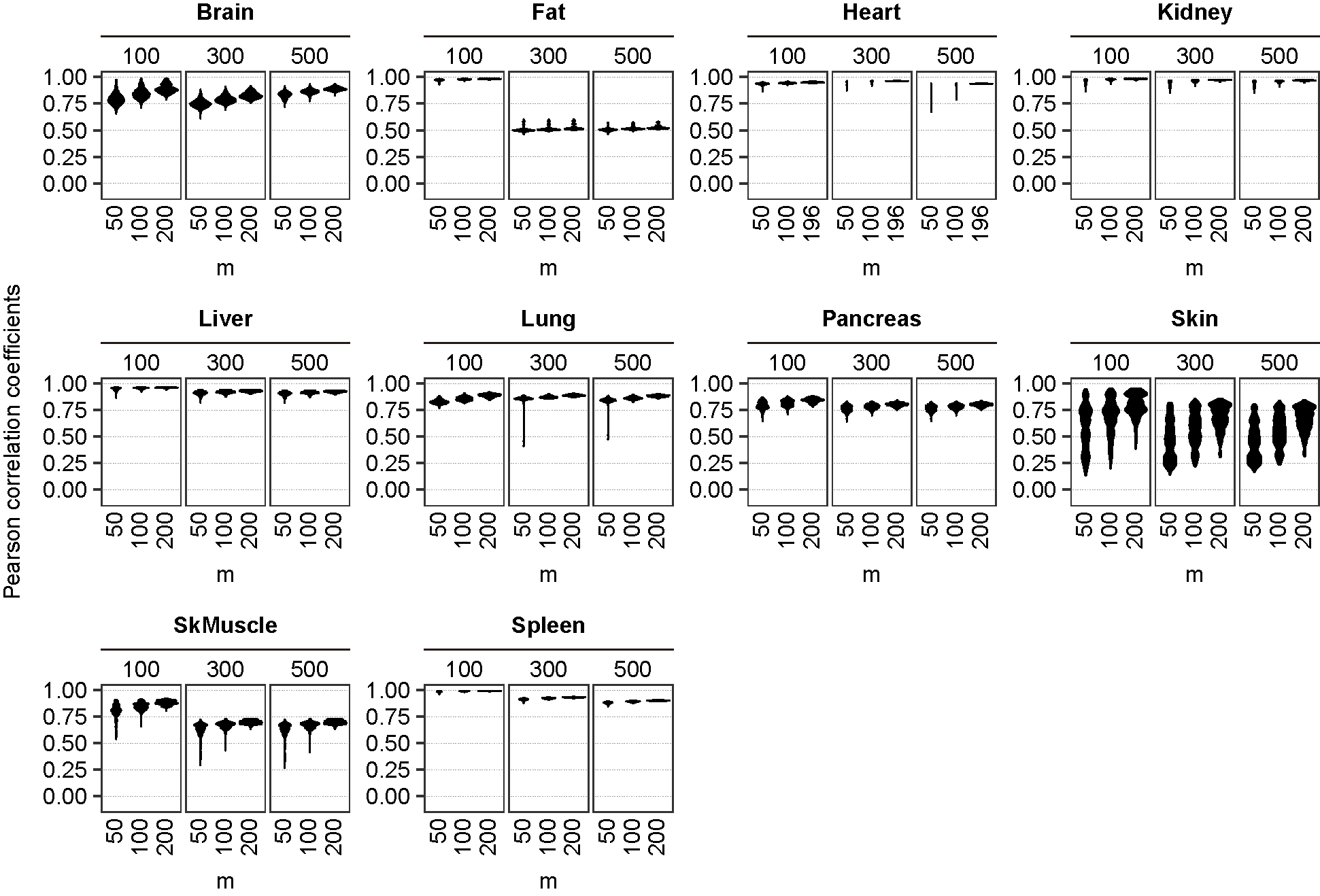

Supplement: S2 Fig — The similarity is shown as violin plot of the Pearson correlation coefficient for each number of the signature genes (100, 300, 500) for each organ (indicated above each plot). The number of m is indicated at the bottom of each graph. The results with the default m = 100 are the same ones shown in Fig 4. Raw data are available as S6 Table. SkMuscle: skeletal muscle. (TIF) [file pgen.1011436.s002.tif]

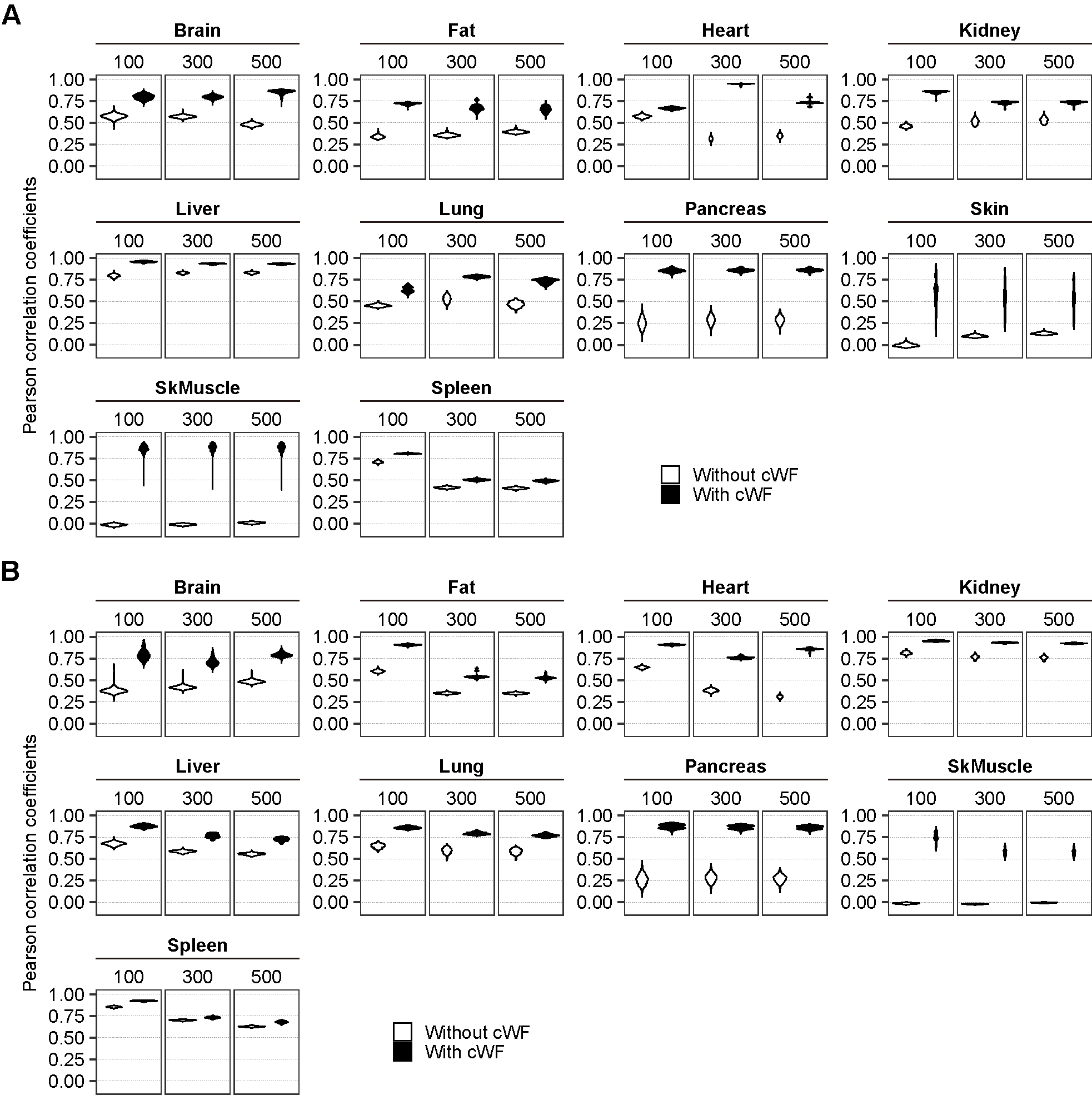

Supplement: S3 Fig — The Pearson correlation coefficients with and without cWFs are shown for each organ using Quant 3’ mRNA-seq (A) (raw data are available as S7 Table) and deep RNA-seq (B) (raw data are available as S8 Table). Independently prepared RNA samples are used for each RNA-seq methods. (TIF) [file pgen.1011436.s003.tif]

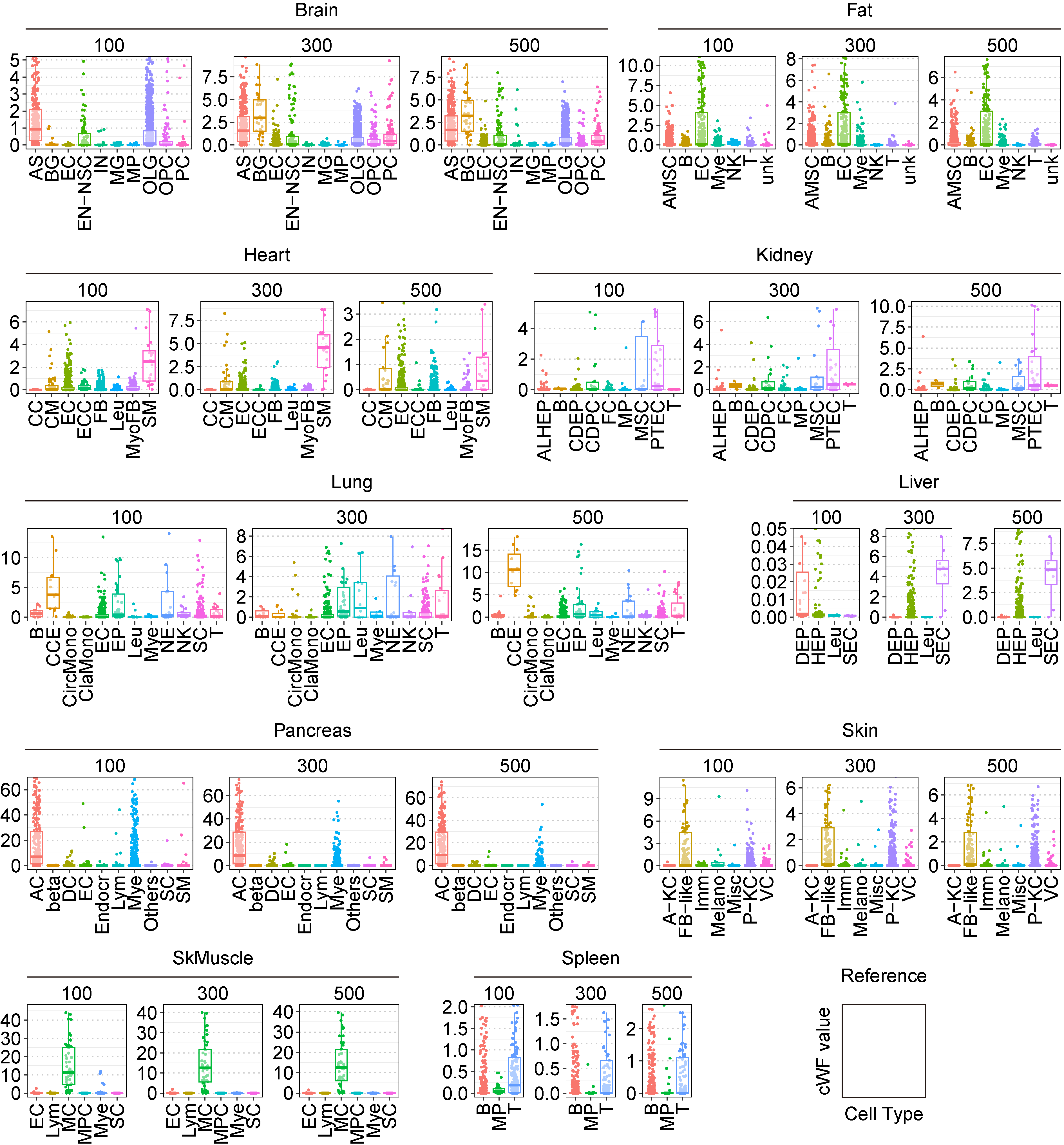

Supplement: S4 Fig — Shown are cWFs for each cell-type of each organ. Compare the results to those shown in Fig 3. Raw data are available as S9 Table. (TIF) [file pgen.1011436.s004.tif]

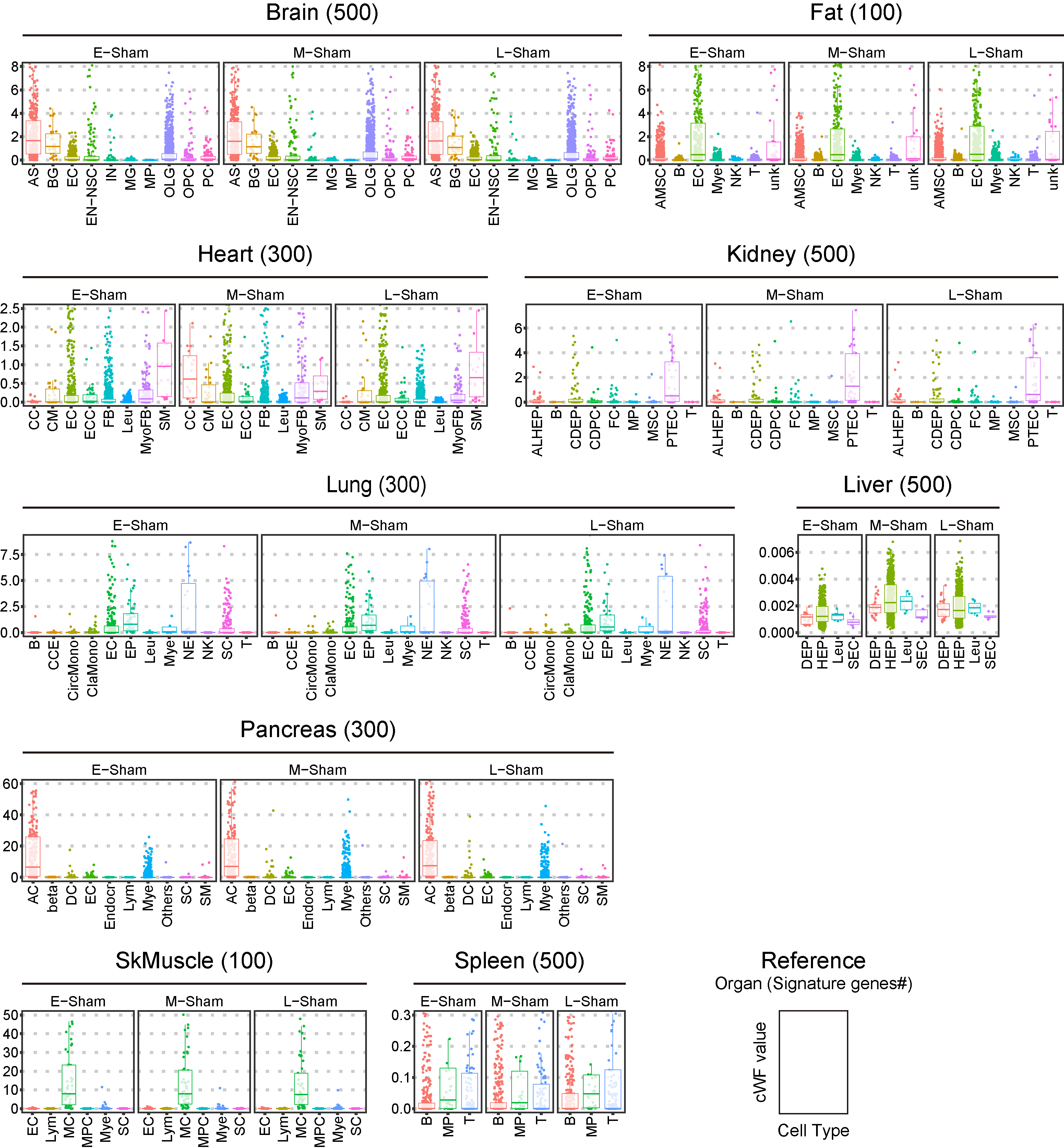

Supplement: S5 Fig — Shown are cWFs for each cell-type of each organ. The sequencing method is the same as those of Fig 3; however, the organ/RNA samples are independently prepared and sequenced. Compare the results to those shown in Fig 3. Raw data are available as S10 Table. (TIF) [file pgen.1011436.s005.tif]

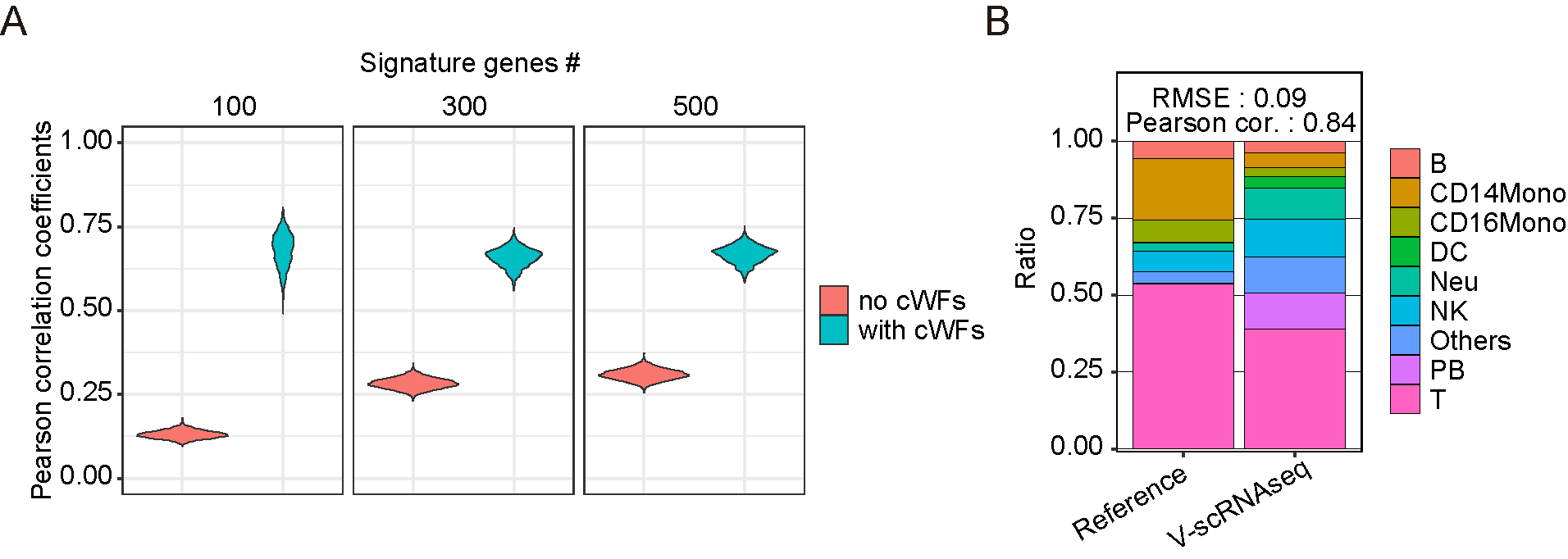

Supplement: S6 Fig — (A) Reconstitution results with and without (no) cWFs are compared. The results with the 100, 300, 500 signature genes are shown. The similarity is shown as violin plot of the Pearson correlation coefficients. The corresponding raw data with no cWFs and with cWFs are found in S14 and S15 Tables, respectively. (B) Bar graph showing the cell type-ratios computed by the deconvolution method (V-scRNAseq) for each organ. The deconvolution was performed with the cWFs computed using the optimal number of the signature genes for each organ (indicated in the accompanying S16 Table, where the best performing result–i.e., the lowest RMSE and the highest Pearson correlation coefficient shown in S6B Fig is highlighted in light green). The bar graphs are composed of the cell-types computed to be present for each organ by our method. The similarity scores (RMSE: Root Mean Squared Errors, Pearson correlation coefficient) are indicated at the top of the bar. (TIF) [file pgen.1011436.s006.tif]

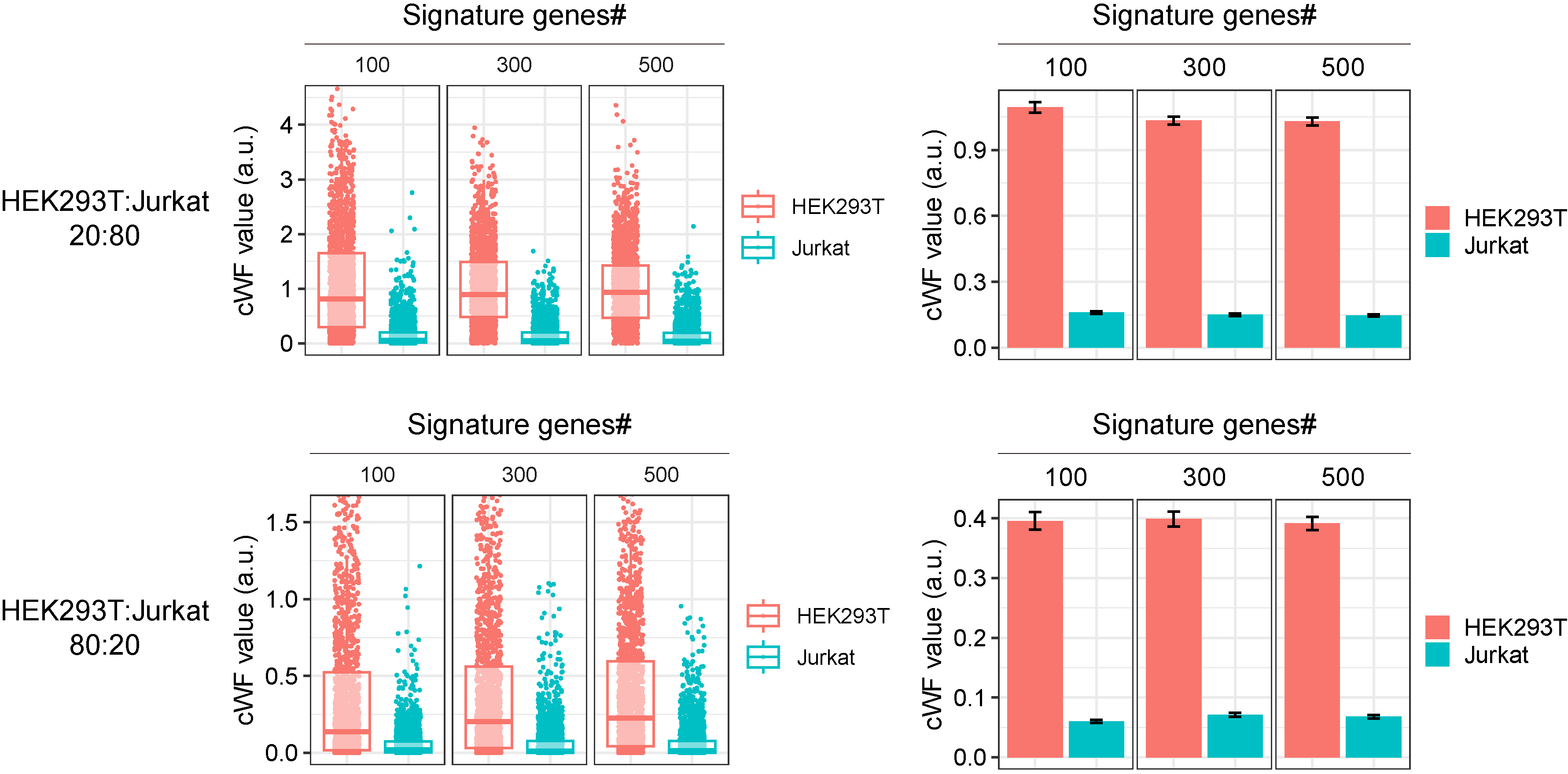

Supplement: S7 Fig — The cWFs are shown for HEK293T and Jurkat cells when they are mixed at 20: 80 and 80: 20 ratios. The results are shown by both box plots (left) and bar graphs (right). The bar graphs are indicated by mean ± S.E. The results with the signature genes number (Signature genes#), 100, 300, and 500 are shown. Raw data are available as S17 Table. (TIF) [file pgen.1011436.s007.tif]

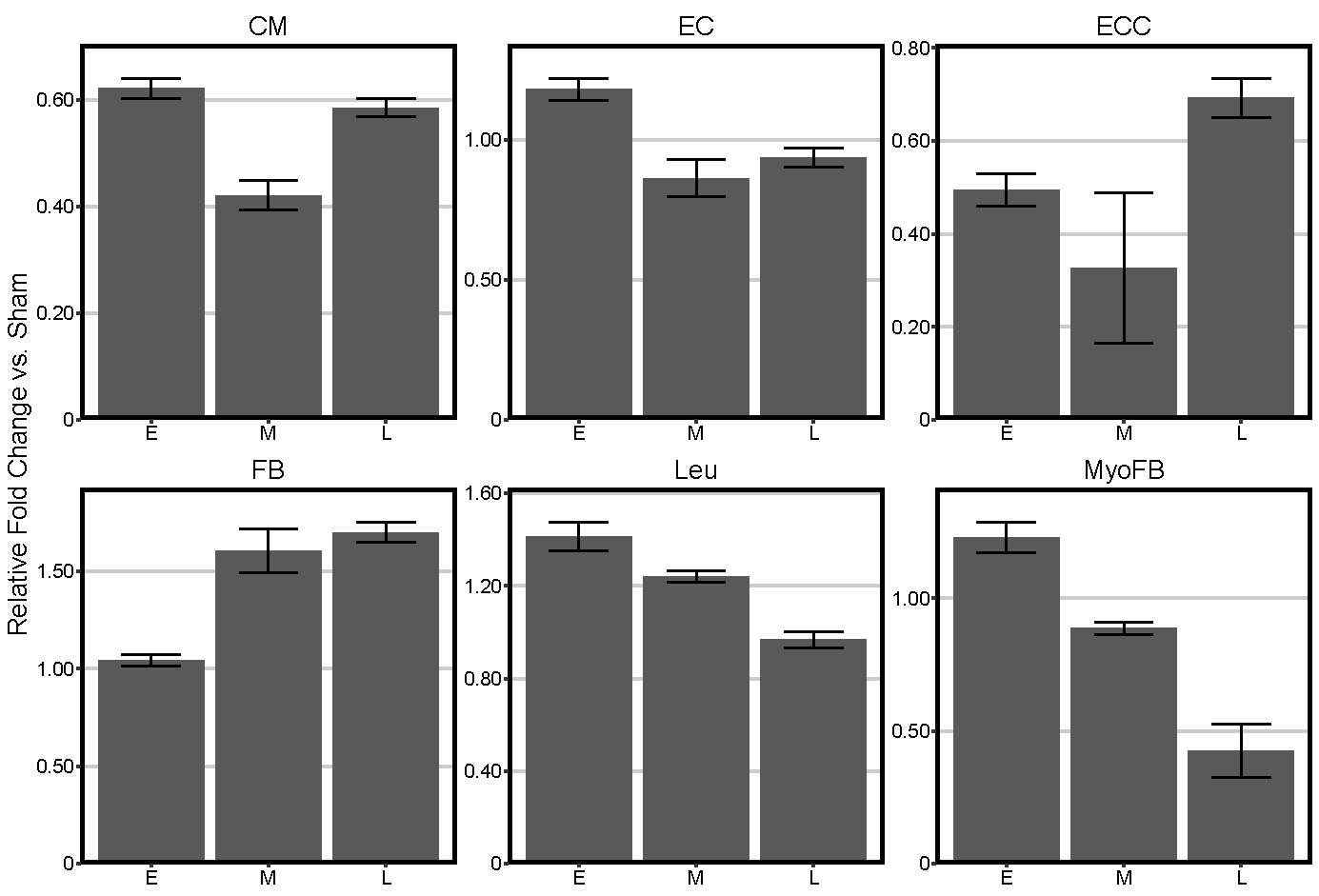

Supplement: S8 Fig — The ratios for each cell-type (CM: cardiomyocyte, EC: endotheial cell, ECC: endocardial cell, FB: fibroblast, Leu: leukocyte, MyoFB: myofibroblast) at each MI stage (E: early MI, M: middle fibrosis, L: late remodeling) are indicated as their relative fold changes to their corresponding sham controls. The MI stages (E, M, L) are defined as previously described [26]. The cWFs were calculated from the sham operated mice data at each stage, using the number of the signature genes, 300, which was optimal in the analyses of deep RNA-seq data from 11 weeks-old male C57BL6/N Jcl mice. These cWFs of the sham operated mice data are used for the deconvolution of the MI data. The bar graphs are shown as mean ± S.E. Raw data are available as S18 Table. (TIF) [file pgen.1011436.s008.tif]

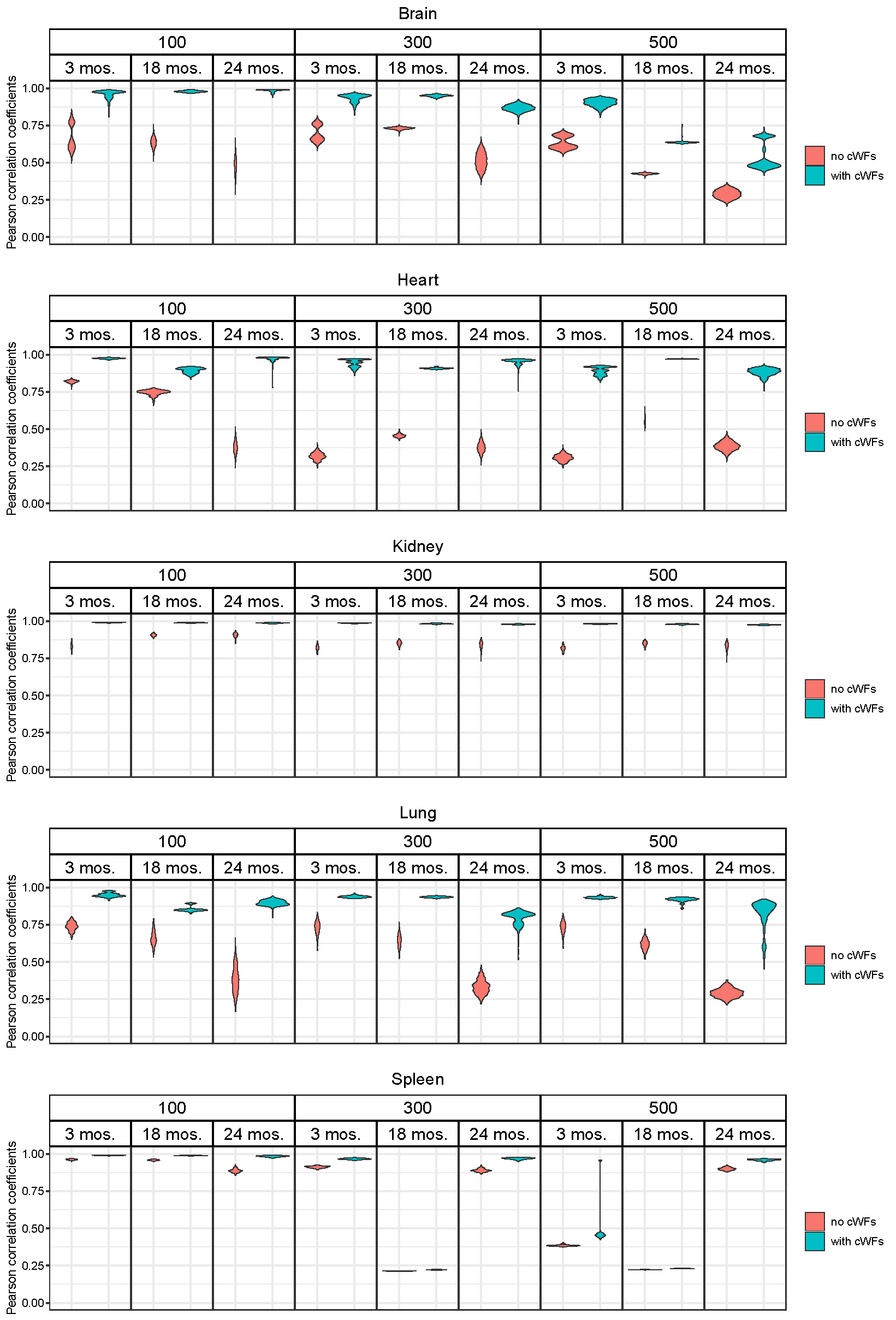

Supplement: S9 Fig — The results with and without (no) cWFs are compared for each aging-stage (3 mos., 18 mos., 24 mos.) for each number of the signature genes (100, 300, 500) and for each organ (Brain, Heart, Kidney, Lung, Spleen). The similarity is shown as violin plots of the Pearson correlation coefficients. The corresponding raw data with no cWFs and with cWFs are found in S19 and S20 Tables, respectively. (TIF) [file pgen.1011436.s009.tif]
