# Supplementary material for: Cell type-specific weighting-factors to solve solid organs-specific limitations of single cell RNA-sequencing
Source: PLoS Genet. 2024 Nov 18;20(11):e1011436. doi: 10.1371/journal.pgen.1011436 (PMC11573148; doi:10.1371/journal.pgen.1011436)
Supplement: Attachment — Submitted filename: Point-by-point_response_PGENETICS-D-24-00035_final.pdf [file pgen.1011436.s033.pdf]

Manuscript ID: PGENETICS-D-24-00035

Point-by-point responses to reviewers.

First of all, we thank the two reviewers for her/his constructive comments and suggestions. We really appreciate for these and we believe that we addressed all her/his comments and suggestions in our revised manuscript. As a consequence, we are delighted to see much improved manuscript. We summarize our "point-by-point response (non-italicized) to each of their comments and suggestions (***bold/italicized***) below that are all reflected in our revised manuscript.

Reviewer #1:
In this manuscript, the authors introduce a novel metric called the "cell-type-specific weighting-factor (cWF)" to address a limitation in current single-cell RNA-seq technology. The internal normalization process in the analyses currently used tends to eliminate putative transcriptome size variations among different cells, thereby obscuring potentially significant functional differences. The literature has already highlighted the importance of transcriptome size in cellular analyses. The authors validated the proposed metric through the reconstitution and deconvolution of bulk RNA-seq data and demonstrated its predictive capability for aging progression.
However, I still have some comments below hope to be addressed.

Major Comments

1. The validation of the cWF involves re-construction of the Bulk RNA-seq data from both sc-RNA seq data and estimated cWF (from Figure 1 and Figure 4). The substantial improvements have been observed. However, could the author please clarify whether the estimated Pearson correlation for Figure 4 is from the results of within- sample prediction or out-of-sample prediction? If the results are from within-sample prediction, could the author provide the results from out-of-sample prediction? Would this be a potential way of addressing the limitation of the current method, that is requiring both sc-RNAseq and Bulk RNAseq at the same time? It will be very helpful to set up more discussions.

Thank you very much for the comment. They are indeed "out-of-sample" predictions, i.e., bulk RNA-seq and scRNA-seq databases are derived from independent samples. To make this point clearer, we added S22 Table to describe the sources of the data and in which analysis/analyses they are used in the study. Furthermore, in the revised manuscript, we added two additional examples of "out-of-sample" results as S2A&B Fig.

Accordingly, we revised the corresponding sections on this point as follows:

(Page 15, line 342 – line 344 of the revised manuscript)
The similar improvement was found with additional organ RNA-seq datasets (S2 Fig), further supporting the notion that the method does not necessarily require both whole-organ RNA-seq and the corresponding scRNA-seq data at the same time.

(Page 28, line 662 – line 669 of the revised manuscript)
Another limitation could be the requirement of both bulk and single-cell RNA-seq data of the organs and the tissues of interest. Our method requires both of these datasets for the target organs/tissues to infer the cWFs of the composing cell-types. However, this may become less of a limiting factor as the availability of such data is exponentially growing. Once we determine the cWFs for the specific cell-

types of organs, the same cWFs can be used for the same cell-types of the corresponding organs derived from other independently-generated datasets (i.e., regardless of their sources). Therefore, the cWFs reported in this study can be used for the same 76 cell-types and 10 organs for other studies.

2. Page 9, 162. The reconstruction of the Buk RNA-seq data involved the mean value of cWF for each cell type. However, the distributions of cWF under certain cell types and cancer types are highly skewed and with larger variation, could the author provide more rational of using the mean value of cWF? Could the author conduct some sensitivity analysis to evaluate results using other robust measures, i.e. median?

Our description in the original manuscript was insufficient. We did NOT use the mean value, instead we used the individual cWFs for the calculations. Hence, we revised the description as follows:

(Page 14, line 320 – Page 15, line 333 of the revised manuscript)
First, we validated the cWFs by the reconstitution (Fig 4) (see also "Datasets", "Data preprocessing", "Calculation of reference cell-type ratios", "Selection of signature gene sets" in the previous Description of the Methods section for the details). Using the individual cWFs for each cell-type (i.e., one cWF for each cell subject up to the total number of cell subjects, m), we weighted the transcript counts for each cell-type. We then summed these weighted transcript counts for all composite cell-types of each organ according to their ratios to construct the synthetic whole-organ RNA-seq (syn-woRNAseq(+cWF)), data. Then, they are compared to the corresponding real whole-organ RNA-seq (r-woRNAseq) data. Specifically, in every recursion of computing the cWFs as described in "Computation of cWFs" of the Description of Methods section, Pearson correlation coefficients were calculated between the real whole-organ RNA-seq and the corresponding synthetic whole-organ RNA-seq generated with or without the weighting-factors (Fig 4). The result shows the Pearson correlation coefficients of 0.8 – 1.0 between the syn-woRNAseq(+cWF) and r-woRNAseq for all 10 organs with all or at least one of the 100, 300, 500 signature genes (Fig 4).

(Page 8, line 163 – line 165 of the revised manuscript)
The process of both random-selection of the cell subject combination and $\widetilde{w}_j$ calculation was recursively performed until more than 100 w_j were generated for all selected cell subjects.

3. Page 20, 376, could the author please have more description of the definition of the prediction accuracy? For Figure 9A, could the author provide more discussion of why the prediction accuracy for Spleen is way lower than the rest of the cancer types?

Thank you very much for this comment. In the revised manuscript, we describe the definitions of accuracy, precision, recall, F1 as prediction metrics in the corresponding section as follows:

(Page 26, line 614 – line 622 of the revised manuscript)
The model was generated using the best parameters and evaluated using test data by accuracy, macro-Precision, macro-Recall, and macro-F1_score. These evaluations were performed by a Python package, '*scikit-learn*' [53] and their formulas are as follows:

$$\text{accuracy} = \frac{1}{3}\sum_{a=1}^{3} \frac{TP_a + TN_a}{TP_a + FP_a + TN_a + FN_a} \quad (13)$$

$$\text{macro} - \text{Precision} = \frac{1}{3}\sum_{a=1}^{3} \frac{TP_a}{TP_a + FP_a} \quad (14)$$

$$\text{macro} - \text{Recall} = \frac{1}{3}\sum_{a=1}^{3} \frac{TP_a}{TP_a + FN_a} \quad (15)$$

$$\text{macro} - \text{F1 score} = \frac{1}{3}\sum_{a=1}^{3} \frac{2}{\left(\frac{TP_a}{TP_a + FP_a}\right)^{-1} + \left(\frac{TP_a}{TP_a + FN_a}\right)^{-1}} \quad (16)$$

where, TP_a, FP_a, TN_a and FN_a represent true positive, false positive, true negative, false negative, respectively, in the classification of the aging stage, a.

In the revised manuscript, we also discuss why the spleen prediction performance is way lower than the rest of the organs as follows:

(Page 27, line 635 – line 638 of the revised manuscript)
The spleen shows significantly lower performance (Fig 9). This may imply that the transcriptome-sizes of three cell-types (B cell, T cell, macrophage) of this organ are similar. Alternatively, the prediction performance is lower due to the small number of cell-types features (i.e., three cell-types).

4. Page 27, 569, the total number of cell subjects, m, is arbitrarily set to 100. Would the estimated cWF vary with a different number of m selected? More sensitive analysis is recommended to see how robust the estimation of cWF w.r.t to the total number of cell subjects, m.
Thank you very much for this suggestion. We now performed sensitivity analysis for m by varying the total number of cell subjects and show the robustness of the result in S1 Fig. The result is described in the revised manuscript as follows:

(Page 8, line 176 – line 181 of the revised manuscript)
The default total number of cell subjects, m, is set to 100 in computing the cWFs. Sensitivity analysis for the total number of cell subjects was performed by varying m to 50 and 200 (196 for the heart, as this is the maximal number of synthetic cell subjects allowed in the computation due to the smaller limiting number of real cardiomyocytes (CMs) in the scRNA-seq data of this organ). The results show no significant differences by varying the number of cell subjects (S1 Fig, in comparison to Fig 3).

Minor Comments:

1. Page 12, 194, there should be space between "t"and "=".
Thank you very much for identifying this typo. We corrected it in the revision as follows:

(Page 16, line 363 of the revised manuscript)
For the iteration (t = 1, …, T), ….

2. Page 29, 616, would zero-truncated normal help instead of setting zeros for negative value?
Thank you very much for suggesting us to use zero-truncated normal. We conducted the analysis using zero-truncated normal (Please see the Fig. below):

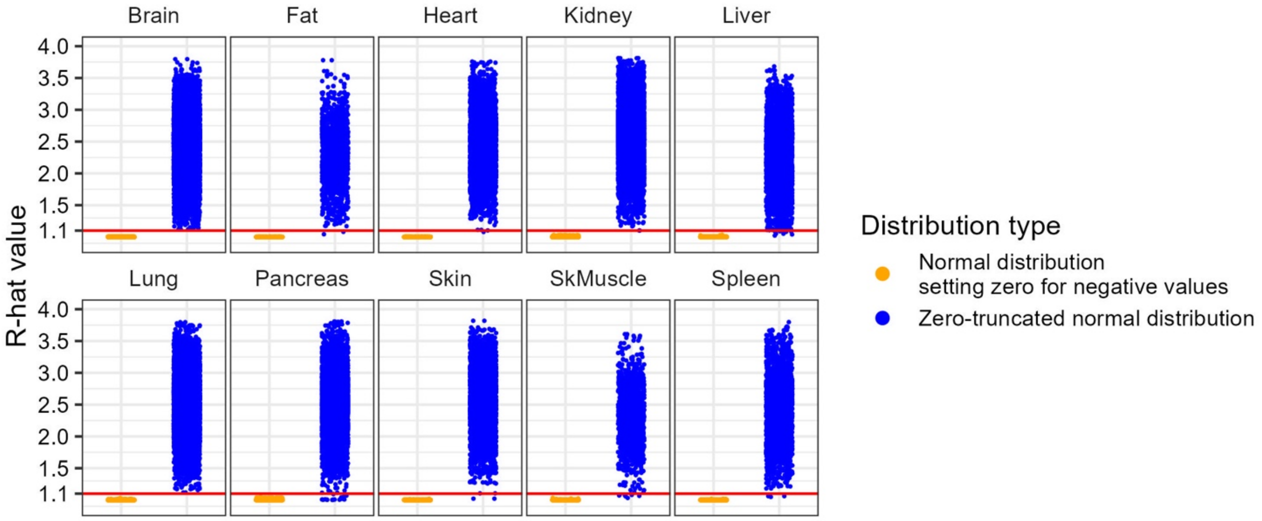

The result shows that using "normal distribution setting zero for negative values" (as in the original manuscript) resulted in successful convergence as indicated by R-hat values below 1.1 for all organs (red). In contrast, using "zero-truncated normal distribution" resulted in failed convergence as indicated by R-hat values above 1.1 for most of the variable/parameters for all organs (blue).

Hence, this result appears to justify the use of "normal distribution setting zero for negative values", instead of "zero-truncated normal distribution".

Reviewer #2

The manuscript titled "Cell type-specific weighting-factors to solve solid organs-specific limitations of single cell RNA-sequencing" introduces cell type-specific weighting-factors (cWFs) to solve the critical limitations of transcription size variability during scRNA-seq analysis. And the authors also performed cWFs to effectively predict aging progression, suggesting the diagnostic applications and association with aging mechanisms. While the concept of cWF is interesting, there are potential limitations in the calculation especially the challenges in distinguishing between the cWF and the cell type population. Detailed comments bellow:

Thank you very much for the valuable comments. We addressed the issue of ***"potential limitations in the calculation especially the challenges in distinguishing between the cWF and the cell type population"*** under each comment below:

1. The weight for transcription size is calculated by solving quadratic problem, which has led to difficulties in distinguishing the weight from the real population percentage for the samples without cell-type ratios. In addition, Since the transcription size for a specific cell type should ideally remain consistent across different conditions, I am wondering if a specific cell type is sharing consistent cWF among different sequencing samples?

This comment is related to the comment 1 of the Reviewer #1 above. Here, we would like to re-emphasize that, based on our algorithm, the absolute cWF values are not determined – instead the "relative" difference in the weights among different cell-types of a particular organ is determined as cWFs. This relative difference in the weights among different cell-types remains consistent for the same organ regardless of the sequencing samples. We show this in S3&S4 Figs in the revised manuscript and the result is described as follows:

(Page 15, line 344 – line 349 of the revised manuscript)
In fact, the relative differences of cWFs among the composite cell-types of each organ remained similar regardless of the organ RNA-seq datasets (S3&S4 Figs, and Fig 3). Only exception is the liver, where the relative differences of cWFs among the liver cell-types appear variable. The liver is predominantly hepatocyte (>95%), suggesting that the method is effective with highly-heterogenous organs, but not with relatively homogenous organs such as the liver.

2. It is important to investigate whether the cell type proportion affects the weight of transcription size. In other words, if the authors simulate bulk RNA-seq data with different compositions of the same cell types, is the cWF changing along with the proportion changes? Furthermore, if the authors use different weights to simulate bulk RNA-seq data, it is essential to assess whether the cWFs algorithm can accurately predict the weight.

Thank you very much for this comment. We addressed this point by varying cell-type ratios and show that the relative cWF difference between the cell-type remains constant. Furthermore, this relative difference of cWFs reflects the known differences of transcriptome-size (i.e., cellular RNA contents) between the cell-types. The result is shown as S6 Fig. in the revised manuscript. The result is described as follows:

(Page 22, line 510 – line 519 of the revised manuscript)
Next, we examined whether differential cell-type ratios influence the cWFs. For this purpose, we computed the cWFs using the bulk RNA-seq data of two cell-types that are mixed to the predetermined differential ratios (S6 Fig) (see also "Datasets", "Data preprocessing", "Selection of signature gene sets" in the previous Description of the Methods section for the details). In this analysis, we used the bulk RNA-seq data derived from the samples where HEK293T and Jurkat cells are mixed to the ratios of 20 : 80 and 80 : 20 [10]. The RNA content of HEK293T cells is determined to be approximately six-times more than that of Jurkat cells [10]. The result shows the expected approximately six-fold larger RNA content of HEK293T cells, as compared to that of Jurkat cells, regardless of their differential ratios (i.e., 20 : 80 vs. 80 : 20) (S6 Fig). This result supports the notion that the cWFs are unaffected by differential cell-type ratios.

The latter part of this reviewer's comment "***if the authors use different weights to simulate bulk RNA-seq data, it is essential to assess whether the cWFs algorithm can accurately predict the weight.***" is interesting – however, it is impossible to experimentally vary the cellular RNA contents without influencing the overall gene expression or cell-type ratios. Instead, we show two examples where the differential transcriptome-sizes are accurately predicated as the relative differences of cWFs among/between the composite cell-types using the corresponding bulk RNA-seq data. The results are shown in Fig. 7 (originally included) and also S6 Fig. above (added in the revised manuscript).

3. In Fig 3, it is observed that cWF values for the majority of cell types are around 0, indicating low transcription size. According to the formula in Fig 2, which states that the transcription size equals scRNA-seq times cWF, this suggests that the majority of these cell types did not express any RNA molecule, which is biologically unreasonable.

Thank you very much for pointing this out and we apologize for the difficulty of seeing the figure, which may have caused some misunderstanding/misinterpretations. As described above for the comment 1, our algorithm determines the "relative" difference of the cWFs among different cell-types of a particular organ, NOT their absolute values. Hence, cWFs are not 0 (zero). Below, we show another version of Fig. 3 where the cWFs are shown on $\log_{10}$-scale, to show that they are small values but NOT 0 (zero).

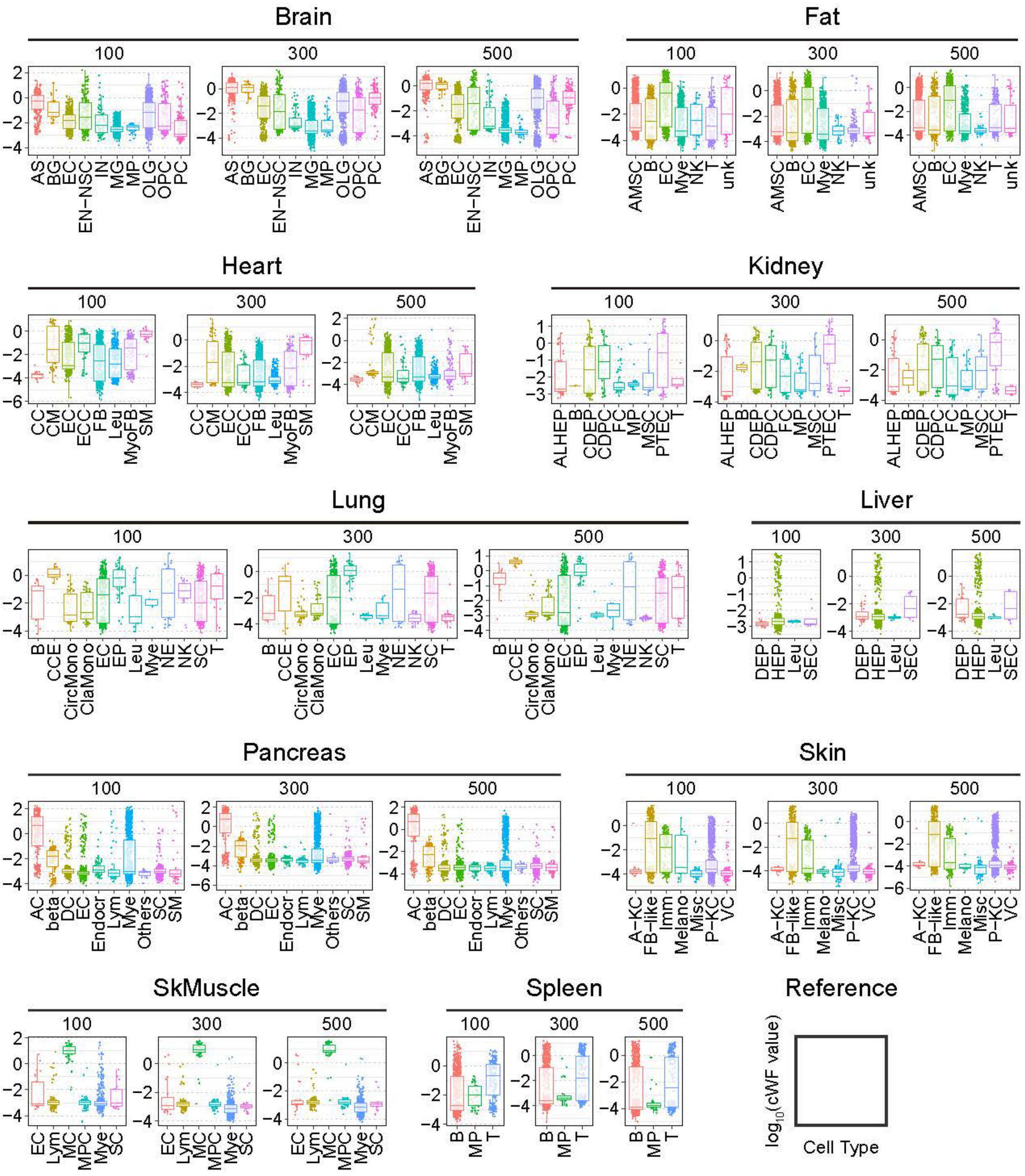

4. It is indicating the cWF calculation require the input of 'celltype_ref_ratio' in the document of github. Then, the requirement for 'celltype_ref_ratio' in the calculation of cWF would introduce bias in the deconvolution process, as the authors utilize the real ratio as the input.

Thank you very much for this valuable comment. We agree that our method is not completely bias-free as the calculation requires the reference cell-type ratios as the input as this reviewer indicates. However, in the revised manuscript, we show an example where "the change" of the cell-type ratio associated with a pathological condition such as myocardial infarction can be predicted despite using the reference cell-type ratio of the healthy condition. The result is shown as S7 Fig. and described in the corresponding sections as follows:

(Page 22, line 523 – Page 23, line 535 of the revised manuscript)
We next evaluated whether our method always require the exactly matched organ (bulk)-RNA-seq and scRNA-seq data of the organ of interest. For this purpose, we performed the deconvolution of the heart RNA-seq data of a myocardial infarction model [26, 47], using the cWFs of the normal heart (i.e., sham control shown in S4 Fig) to compute a putative change of the cell-type ratios of the infarcted heart (S7 Fig) (see also "Datasets", "Data preprocessing", "Calculation of reference cell-type ratios", "Selection of signature gene sets" in the previous Description of the Methods section for the details). The results show that the method can detect the expected cell-type ratio changes [48, 49] – i.e., the significantly decreased cardiomyocytes (CM) at the early MI stage (ca. × 0.62) (E in S7 Fig) and their further reduction at the middle fibrosis stage (ca. × 0.42) (M in S7 Fig), and increased fibroblasts (FB) at the fibrosis (ca. × 1.61) (M in S7 Fig) and late cardiac remodeling (ca. × 1.70) (L in S7 Fig) stages. These results suggest that the deconvolution method herein does not necessarily require the prior knowledge about the exact cell-type ratio of the target organ.

(Page 29, line 674 – line 677 of the revised manuscript)
Furthermore, we show at least one example where the expected pathological changes of the cell-type ratio of the heart can be predicted using the cWFs derived from the healthy control heart (S7 Fig), despite a bias introduced by this reference data.

5. The authors utilized cWF for deconvolution in Fig 5. As shown in Fig 3, the SEC in the liver exhibits a higher cWF value than other cell types, indicating that the SEC has a larger transcription size and would reduce the percentage of SEC during deconvolution. However, when compared with the true percentage, DWLS and MuSiC results, the SEC cell type composition increases in cWF. This discrepancy between the cWF and deconvolution results presents a controversial aspect.

Thank you very much for pointing this out. Despite the effective and improved predictions for many organs, the prediction performance of the liver is unsatisfactory. We believe this is in part due to the fact that the liver is predominantly hepatocytes. The prior distribution of our method is set for symmetric Dirichlet distributions among cell-types of the target organs, hence, it is effective for the organs of highly heterogeneous. However, it does not work well with the organ consisting of one predominant cell-type such as the liver where the hepatocyte consists of over 95% of the liver and the other cell-types such as SEC, Leu, DEP, all combined consist of less than 5%. Despite such limitation, we believe the method is useful for many other organs of highly heterogenous cellular composition – in fact, the deconvolution, in most of the situations, is applied to organs of highly heterogenous compositions, rather than to those of predominantly single cell type. We elaborate this point in the Discussion section of the revised manuscript as follows:

(Page 28, line 659 – line 662 of the revised manuscript)
Despite the usefulness of cWFs, some limitations must be noted. While our method is effective for the organs consisting of heterogeneous cell-types, it is ineffective for a relatively homogenous organ such as the liver consisting of predominantly hepatocytes (>95%) (Fig 3 and S3&S4 Figs, Fig 5).
