# Supplementary material for: Cell type-specific weighting-factors to solve solid organs-specific limitations of single cell RNA-sequencing
Source: PLoS Genet. 2024 Nov 18;20(11):e1011436. doi: 10.1371/journal.pgen.1011436 (PMC11573148; doi:10.1371/journal.pgen.1011436)
Supplement: Attachment — Submitted filename: Point-by-point_response_PGENETICS-D-24-00035_r2_final.docx [file pgen.1011436.s034.docx]

**Manuscript ID: PGENETICS-D-24-00035**

**Point-by-point responses to reviewers.**

First of all, we thank the two reviewers for her/his evaluating our revised manuscript and the Reviewer #2 for approving our revision for the publication. Reviewer #1 raised one additional point. We really appreciate for this by Reviewer #1 and we addressed this point in this second revision. We summarize our “point-by-point response (non-italicized) to this comment (***bold/italicized***) below that is reflected in our revised manuscript:

***Reviewer #1:
Reviewer #1: Thank you for addressing my comments comprehensively. However, I have one additional comment that requires your attention.***

***Major Comments:***

***1. In response to the previous major comment 4, you have included a sensitivity analysis to evaluate the estimation of cWF with respect to different numbers of cell subjects, m. However, in Figure S1, the magnitude of the estimated cWF varies significantly with different choices of m, even under the same cancer type and with the same number of signature genes. Could you please provide a more detailed explanation for this variation?***

Thank you very much for the comment and pointing this out. In the revision, we describe the observed variation of the cWFs and also provide an explanation of this variation in the revision as follows:

(Page 8, line 180 – Page 9, line 192)

The results show that the overall relative pattern of the differential cWFs across the composite cell-types of each organ for the same signature gene numbers remain largely unchanged (S1 Fig, in comparison to Fig 3). However, we observed noticeable variations of the cWF values for the same cell-types with differential $m$. In particular, their values appear to decrease with the increasing $m$ for the same cell-types of the same organ. One possible explanation of this result is marginal performance of the $m$ multiplication on the left side of Equation (1) $m\mathbf{y=}\sum_{\boldsymbol{j}} w_{\boldsymbol{j}}\mathbf{X}_{\boldsymbol{j}}\boldsymbol{(}j \in C^{m})$ where $w_{j}$ (cWF per cell) is calculated as a linear regression problem with the constraint of $w_{j} \geq0$ (see Materials and methods). In this equation, the $m$ multiplication on the left side corrects otherwise the reduction of $w_{j}$ with increasing $m$. For example, when this correction function is absent (i.e., $\mathbf{y=}\sum_{\boldsymbol{j}} w_{\boldsymbol{j}}\mathbf{X}_{\boldsymbol{j}}\boldsymbol{(}j \in C^{m})$, the increasing numbers of $\mathbf{X}_{j}$ are used with increasing $m$ for the fixed vector $\mathbf{y}$; hence, the $w_{j}$ is reduced. Therefore, it is possible that the performance of this function is marginal due to yet undefined causes.

***Additionally, how might this variation affect the accuracy of reconstituting the whole organ RNA-seq?***

We evaluated a possible impact of this variation on the whole-organ RNA-seq reconstitution. The result is shown as new S2 Fig and Table #6 in the revision. The result is also described in the text as follows:

(Page 15, line 353 – line 357)

Despite the variations of the cWF values induced by the differential $m$ (S1 Fig, Fig3), the reconstitution with these varying cWFs shows similarly significant improvement to that without cWFs (S2 Fig, compare to Fig 1). One noticeable difference is that the increase of $m$ appears to slightly improve the Pearson correlation coefficients (S2 Fig), suggesting a benefit of increasing $m$.

***Reviewer #2
Reviewer #2: The authors have addressed all comments and questions effectively, and the cWF has proven to be a great tool for evaluating transcription size for different cell types. I recommend accepting this paper as it is suitable for publication in PLOS Genetics.***

Thank you very much for approving our revision and recommending the acceptance for its publication in PLOS Genetics.
